# Welfare indicators in cattle farming in the face of heat stress: a review in climate change scenarios

**DOI:** 10.3389/fvets.2025.1754412

**Published:** 2026-02-11

**Authors:** Italo Messias Ferreira de Souza, Carlos Eduardo Lima Sousa, Vanessa Sousa Pinto, Luís Gustavo Paixão Vilela, Alinne da Silva Souza, João Paulo de Sousa Cunha, Cláudio Vieira de Araújo, Marina de Nadai Bonin Gomes, Lílian Kátia Ximenes Silva, Lucietta Guerreiro Martorano, Kedson Alessandri Lobo Neves, Raimundo Nonato Colares Camargo-Júnior, Éder Bruno Rebelo da Silva, Welligton Conceição da Silva

**Affiliations:** 1 Postgraduate Program in Animal Science, Institute of Agricultural and Environmental Sciences, Federal University of Mato Grosso (UFMT), Cuiabá, MT, Brazil; 2 Academics of the Veterinary Medicine Course at the University Center of the Amazon (UNAMA), Santarém, PA, Brazil; 3 Postgraduate Program in Animal Science, Federal University of Mato Grosso do Sul, Campo Grande, MS, Brazil; 4 Federal University of Pará (UFPA), Castanhal, Pará, Brazil; 5 Embrapa Eastern Amazon, Santarém, PA, Brazil; 6 Federal University of Western Pará (UFOPA), Santarém, Pará, Brazil; 7 Federal Institute of Education, Science and Technology of Pará (IFPA), Santarém, PA, Brazil; 8 Lutheran University Center of Santarém (ULBRA), Santarém, PA, Brazil

**Keywords:** adaptation, animal, bioclimatology, climate, environment, thermoregulatory mechanisms

## Abstract

This work consists of a narrative review that addresses the differences between European cattle and Zebu cattle in their resilience to environmental challenges. It was developed based on scientific articles, theses, dissertations, and technical documents available in recognized databases such as Web of Science, ScienceDirect, Scopus, and PubMed, prioritizing recent studies from 2020 to 2025 that are relevant to the topic. The method used was a narrative review, in which publications addressing the physiological, behavioral, bioclimatic, and adaptive production parameters of each animal group were selected, allowing for a comparative analysis of their main characteristics. The results indicate that European cattle, although highly productive, are less adapted to heat, while zebu cattle stand out for their hardiness, resistance to high temperatures, and lower incidence of diseases. The conclusion is that analyzing these differences is essential to guide breed selection, genetic improvement strategies, and the adoption of more sustainable production systems, favoring greater livestock efficiency and resilience under diverse environmental conditions.

## 1 Introduction

Cattle welfare is a central issue in modern livestock production, particularly in the context of increasing demands for sustainability, efficiency, and resilience of production systems (1, 2). Among the factors influencing animal welfare, the climatic environment plays a decisive role, directly affecting comfort, health, and productive performance (3). Environmental variables such as air temperature, relative humidity, solar radiation, and wind speed, when analyzed in an integrated manner through indices such as the Temperature and Humidity Index (THI), allow a more consistent assessment of thermal conditions experienced by cattle (4).

The evaluation of cattle welfare under climatic conditions is based on physiological, behavioral, productive, and bioclimatic indicators (5). Changes in physiological responses and behavior, as well as variations in productive performance, are widely used to identify heat stress situations and their consequences on animal welfare (6–11). The combined interpretation of these indicators enables the identification of critical thermal stress scenarios that compromise both animal performance and welfare status (12).

Bioclimatic indices have been widely applied to quantify the impact of environmental conditions on cattle thermal comfort, allowing the characterization of heat stress in different production systems (13, 14). Among the most commonly used indices are the Temperature and Humidity Index (THI), Black Globe Temperature and Humidity Index (BGHI), Benezra Thermal Comfort Index (BTCI), Environmental Stress Index (ESI), Equivalent Temperature Index (ETI), Iberian Heat Tolerance Index (IHT), and Thermal Load Index (TLI) (4, 15).

Despite the extensive body of research addressing physiological, behavioral, productive, and bioclimatic responses to heat stress, the available literature remains fragmented, with most studies analyzing these indicators in isolation (6, 16–18). This fragmentation limits a comprehensive understanding of the interactions among indicators and their combined implications for cattle welfare, especially under conditions of climate change and increasing production intensification. In this context, an integrated synthesis of recent scientific evidence is necessary to support decision-making in animal management and thermal stress mitigation strategies (14, 19–22).

The hypothesis is that the integration of physiological, behavioral, and productive indicators allows for a more accurate assessment of the welfare of cattle under heat stress. It is also assumed that bioclimatic indices differ in sensitivity and applicability between environmental conditions and production systems, influencing their efficiency in identifying heat stress. Finally, it is postulated that the incorporation of technological monitoring tools improves the assessment of heat stress and guides more efficient management strategies in climate change scenarios.

Therefore, this manuscript proposes a integrated synthesis of studies published between 2020 and 2025, incorporating technological advances applied to environmental monitoring and thermal management of cattle in tropical and subtropical regions. Thus, the objective of this study was to conduct an integrated analysis of livestock welfare indicators in the face of heat stress: a review in climate change scenarios.

## 2 Materials and methods

For the elaboration of this review, a bibliographic survey was carried out in the Web of Science, ScienceDirect, Scopus, and PubMed databases, which are scientific databases capable of storing high-quality articles. The following keywords were used: “animal welfare indicators,” “heat stress in cattle,” “animal bioclimatology,” and “cattle farming and production systems.”

The inclusion criteria were complete texts, written in English, Portuguese, and Spanish, and that presented a discussion on animal welfare indicators related to the climate environment. Articles were excluded when they did not fit the objective of the study. In this study, we chose to carry out a time frame from 2020 to 2025 and not follow a geographical order during the searches.

The studies were separated by title and abstract, after which they were analyzed in quadruplicate by the authors independently, avoiding interference. The variables retrieved and analyzed from the manuscript were the year of publication of the study, title, species, breed, number of animals in the study, experimental design, technologies applied, and country.

Thus, all available literature was considered until the conclusion of the database searches. A total of 158 references were cited in this review. In this study, the data were organized in spreadsheets and then evaluated, being presented in formats of tables and graphs to favor the reader's understanding. The same methodology was used in the studies by Mota-Rojas et al. (23), Rodrigues et al. (24), Ghezzi et al. (25), and Mota-Rojas et al. (26).

**Figure 1:**
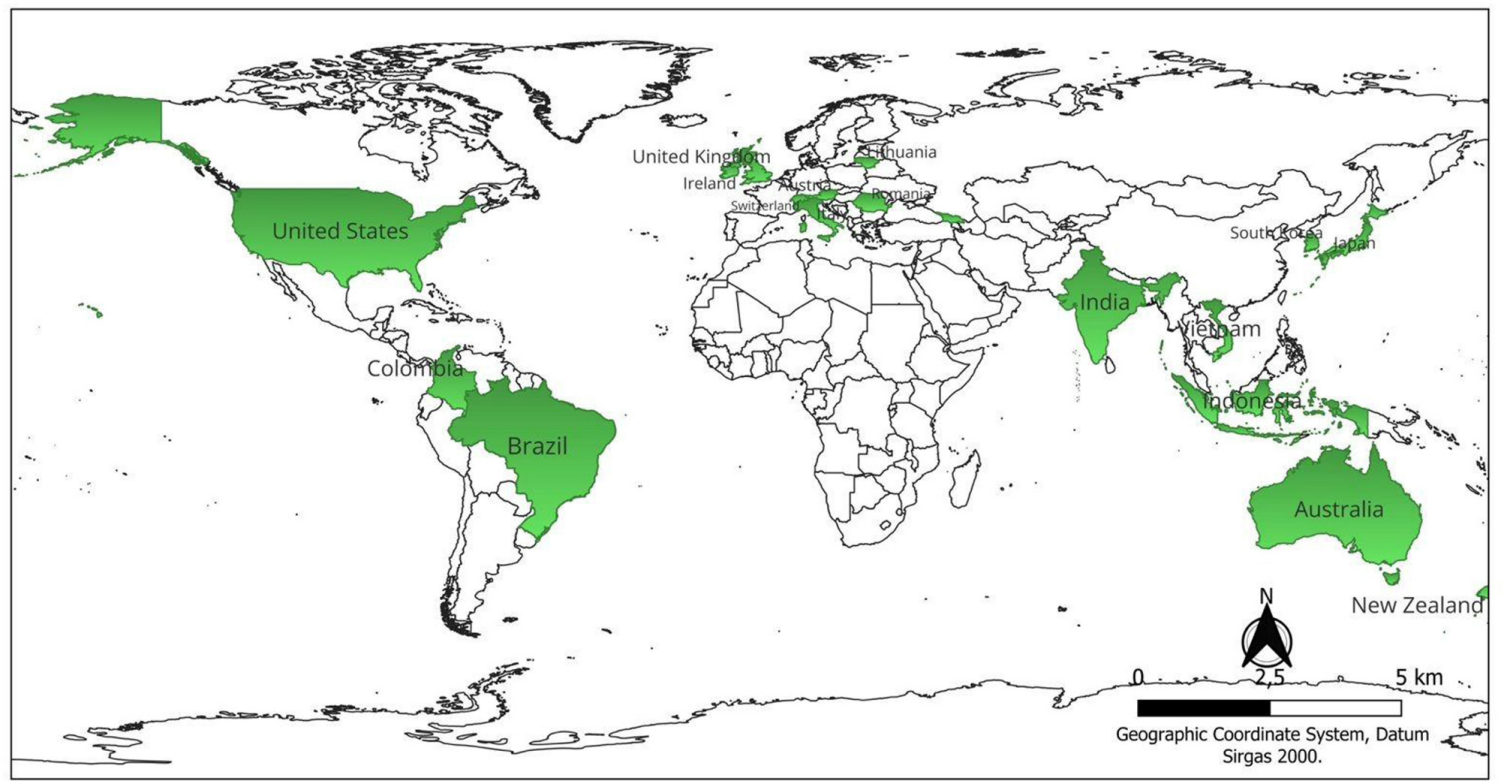
Map of the countries found in the database platform searches and used in this review. Authorship (2025).

Figure 1 shows the countries that have researched animal welfare indicators related to the climate environment. Supplementary Table S1 shows the different experimental studies carried out by adding the year of publication of the study, title of the article, species, breed, number of animals in the study (NA), experimental design and the technologies applied according to Page et al. (27).

## 3 Literature review

### 3.1 Welfare in cattle farming

Animal welfare is one of the central pillars of contemporary cattle farming, being recognized as a fundamental requirement for the sustainability, productivity, and quality of products of animal origin (28). Its importance transcends ethical aspects, encompassing economic, environmental, and social factors, which determine the competitiveness of the sector in the face of increasingly demanding consumers (29). The World Organization for Animal Health defines welfare as the state in which the animal is healthy, well-nourished, comfortable, capable of expressing its natural behavior and free from pain, fear or suffering, consolidating itself as an international reference parameter (14, 16, 18).

The consumer market attributes increasing value to management practices that promote animal welfare, valuing products from ethical and sustainable systems (30). Such demand encourages the adoption of protocols and certifications, such as Welfare Quality^®^, which evaluates wellbeing based on objective indicators related to health, nutrition, environment, and behavior (31). In this way, wellbeing becomes not only a moral issue, but also a competitive advantage and a requirement to meet the demands of the consumer market (16, 28, 32).

The assessment involves multiple factors, including nutrition, health, environment, freedom of movement, and emotional states (33). Animals exposed to stress, fear or discomfort present physiological and behavioral changes that compromise productive performance, reproduction and the quality of meat and milk (34, 35). Thus, the promotion of favorable environments and the adoption of adequate management are essential strategies to balance productivity and animal comfort (36).

Nutrition is one of the main points in maintaining wellbeing, since balanced diets ensure adequate physical development, greater resistance to disease, and stress reduction (37). In addition, the available space and the conditions of physical comfort are determinant, overcrowded or inadequate environments increase the incidence of diseases and reduce productive performance, while facilities that offer ventilation, lighting, thermal comfort and access to clean water favor the health and natural behavior of the animals (17, 38, 39).

Production systems in cattle farming can be classified as extensive, semi-intensive, and intensive, according to the technological level and infrastructure employed (3). The extensive system is characterized by natural pastures, low stocking density and less human intervention, with reduced costs, but variations in productivity (40). The semi-intensive integrates grazing and strategic feed supplementation, promoting nutritional stability and greater production regularity (24). Intensive systems, on the other hand, involves confinement and balanced diets, allowing greater control of production, but with possible impacts on wellbeing due to space constraints (30, 32, 41).

Globalization and the demand for ethical standards drive the standardization of handling, transportation, and slaughter protocols, aiming to reduce economic losses and ensure efficiency throughout the production chain (32). At the same time, society demands transparency and ethical responsibility in dealing with animals, reinforcing the importance of practices that respect welfare (42). The Brambell report (43), a historical landmark of the concept, already highlighted basic recommendations such as adequate space, access to water and quality food, principles that remain central to modern livestock.

In the scientific realm, the assessment of wellbeing has evolved from simply preventing negative experiences to valuing positive states and opportunities for behavioral expression (44). According to Barreto et al. (45), Pichlbauer et al. (46), and Singaravadivelan et al. (47), prolonged impairment of wellbeing, as occurs in inadequate facilities, is more harmful than isolated episodes of acute pain. Thus, the current challenge is to develop indicators that measure not only the absence of suffering, but also the presence of positive experiences, consolidating a comprehensive approach (15).

### 3.2 Influence of the climatic environment

The environment plays a decisive role in the performance and wellbeing of farm animals, especially in tropical and subtropical regions, where temperature, humidity and radiation fluctuations are intense (48). In practice, many producers face an aggravating factor: the removal of native vegetation for the implementation of pastures and forage crops, which drastically reduces the supply of natural shade, exposing the animals to greater thermal loads and reducing behavioral options for heat relief (49). When adaptive capacity is exceeded, heat stress sets in, with negative consequences for health, reproduction, and productivity (50).

The relationship between climate and performance is complex and strained by the economic limitations of the producer (51). High temperatures and high humidity reduce feed intake and alter nutritional requirements, which translates into lower weight gain and production, however, supplementation to compensate for these losses implies additional costs that not all systems can support (52). In many family farms, the difficulty in accessing financial resources for the acquisition of concentrates, storage, and transportation of inputs aggravates productive vulnerability in the face of prolonged heat waves (53).

**Figure 2:**
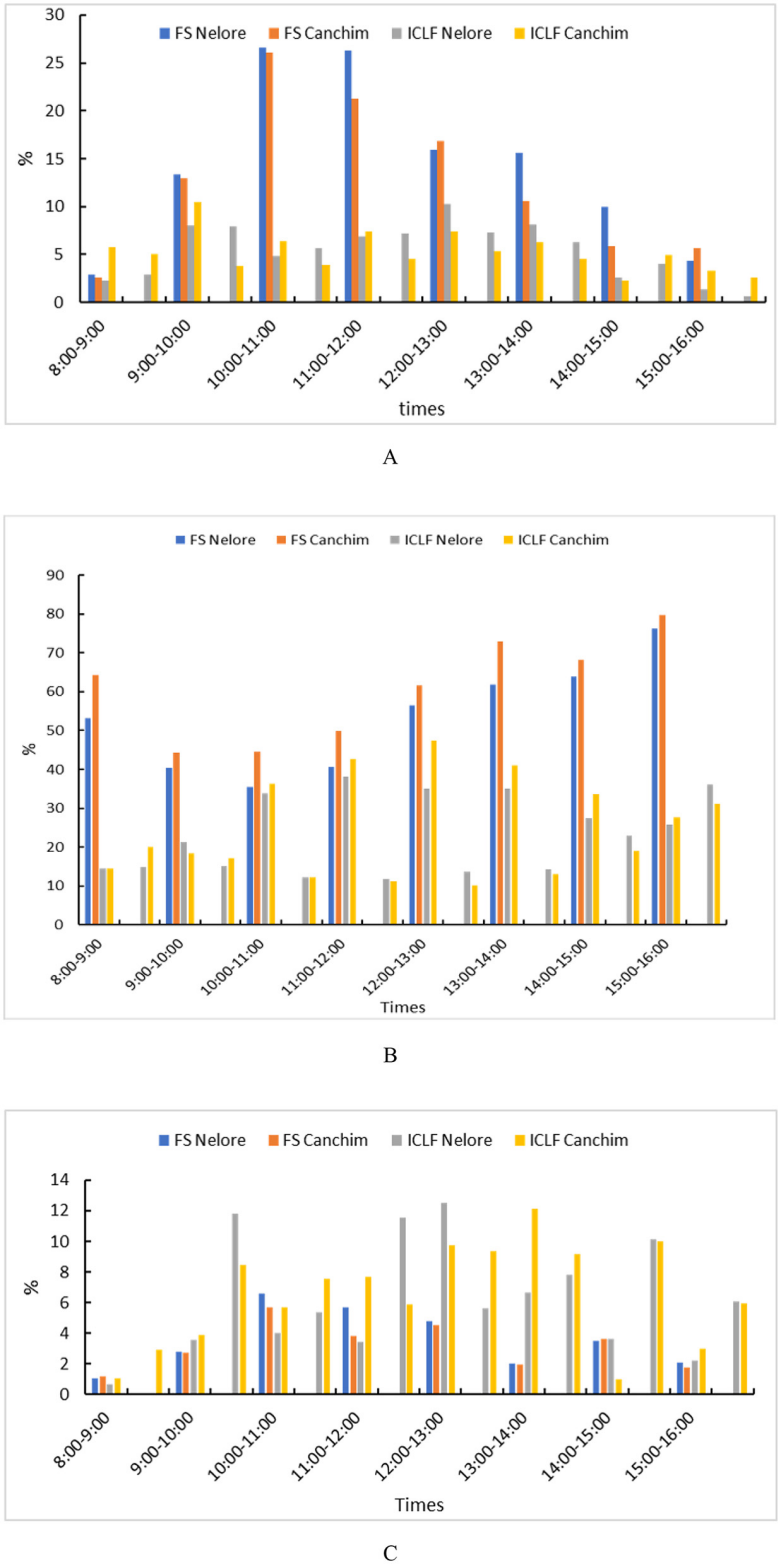
Behavior of young Nellore and Canchim bulls kept in pasture with full sun or in integrated crop-livestock-forest systems. **(A)** Time spent resting lying down; **(B)** Time spent grazing; and **(C)** Time spent ruminating lying down. Adapted from Moraes et al. (40). Full sun (FS) and integrated crop-livestock-forestry (ICLF).

Behaviorally, cattle resort to responses such as seeking shade, reducing activity, and increasing respiratory rate to dissipate heat, however, these strategies are ineffective when shade is nonexistent or insufficient (54). The construction of sheds, installation of mechanical ventilation, sprinkler systems and other cooling devices require initial investment and operating expenses (energy, maintenance), which limits their adoption, especially in small properties (55). Thus, the socioeconomic conditions of the producer directly influence the ability to mitigate climate impacts (Figure 2) (56).

Given this situation, animal bioclimatology provides valuable tools to identify thermal risks and prioritize interventions, however, implementation must consider technically effective and financially viable alternatives (4, 26). Low-cost and high impact strategies—such as shading by tree corridors, rotational management to preserve forage cover, improvement of drinking points and adjustment of management and feeding schedules—associated with genetic selection by more adapted animals, rural extension programs and financing policies, are practical measures that increase the resilience of production units and promote comfort in real production contexts (14).

### 3.3 Climate-related animal welfare indicators

Comfort in conditions of heat stress can be evaluated based on indicators that modify climatic factors, such as temperature, humidity, solar radiation, and wind speed, into signals that reflect the actual condition of cattle (57). These indicators allow us to understand how the environment interferes with health, comfort, and productive performance, and are used as practical tools to guide management (58). For greater accuracy, it is important to associate environmental measures with responses observed directly from the animals, ensuring faster diagnoses and effective interventions (59).

The choice of indicators varies according to the objectives of each property and the reality faced by each producer. It can be used with the aim of prevention, using environmental indices as a “risk alert” (60–62). In other situations, the focus is on diagnosis, using physiological or productive signs to assess the severity of the problem (63, 64).

In the daily life of farms, the value of indicators lies in the ability to transform data into practical and accessible decisions (3, 14, 65). The Temperature and Humidity Index (THI), for example, is widely used as a heat risk benchmark, while observing changes in behavior, such as increased shade-seeking or changing rumination pattern, allows for rapid interventions (63, 66). Physiological parameters, such as respiratory rate or body temperature, and productive parameters, such as a drop in food intake and reduced milk production, help to measure the intensity of heat stress (64, 67).

However, many producers face obstacles that make it difficult to apply these indicators in practice. The absence of tree cover in pasture areas, a result of deforestation for the implementation of forage monocultures, drastically reduces the availability of natural shade (68). Likewise, the limitation of financial resources restricts the installation of sheds, ventilation or cooling systems, making it impossible to adopt more advanced technologies in small and medium sized properties (69). This scenario highlights the need to adopt a tiered approach, prioritizing low-cost and higher impact measures (56).

From this perspective, the integration of simple and accessible alternatives, such as the use of trees for shading, rotational management of pastures and adjustments in the times of food supply, which significantly reduce the effects of heat on cattle (68, 70). These practices can be complemented, whenever possible, by more sophisticated technologies, such as thermal monitoring sensors, infrared thermography, or cooling systems, which allow for greater accuracy in diagnosing and controlling discomfort (26, 71). Thus, the balance between economic feasibility and technical effectiveness becomes essential to promote animal welfare and the sustainability of production systems (14, 67).

#### 3.3.1 Physiological and hormonal indicators

Direct measures of cattle response to heat stress are considered, reflecting changes in the respiratory and circulatory systems (59, 72). The most commonly used physiological indicators are heart rate, respiratory rate, rectal temperature, and surface temperature, which increase proportionally to prolonged exposure to high environmental temperatures. In addition, we associated physiological parameters with hormonal indicators, which are Cortisol, insulin-like growth factor 1 (IGF-1), T3, T4, Glucose, and non-esterified fatty acids (NEFA), helping to assess the wellbeing of cattle (Table 1) (73, 74).

**Table 1:** Physiological and hormonal indicators.

**Indicator**	**Physiological mechanism/ relationship with stress**	**Reference values**	**Stress/alteration**	**Measurement method**	**Limitations**	**Citations in papers**
**Physiological**						
Heart rate	Increased cardiac output and peripheral vasodilation under heat or pain	60–80 bpm	>90 bpm (stress/pain/heat)	Stethoscope, monitors, sensors	Physical exertion and influenced by management, excitement	(5, 6, 14, 56, 75, 79, 93)
Respiratory rate	Ventilation lift to dissipate heat via evaporation	24–36 breaths/min	60–80 breaths/min (discomfort); >100 breaths/min (severe)	Visual counting, respiratory sensors, video/algorithms	Influenced by exercise, arousal; laborious manual counting	(4, 7, 56, 64, 75, 79, 82)
Rectal temperature	Reflects central thermal load; increase indicates thermal stress	38.0 °C−39.3 °C	>39.5 °C (hyperthermia); >40 °C (intense stress)	Rectal thermometer, internal sensors	Rectal = invasive/point; requires interpretation by schedule/water intake	(3, 4, 14, 56, 67, 75, 96)
Surface temperature	Evidence of blood redistribution for heat dissipation	34 °C−36 °C	>38 °C (acute stress)	Infrared thermography (eye/eye contour, face, tail base, udder)	Sensitive to solar radiation, wind, humidity, distance	(4, 14, 67, 78, 81, 86)
**Hormones**						
Cortisol	Activation of the HPA axis; mobilizes energy; alters behavior and immunity	1–6 μg/dl	>10 μg/dl (estresse agudo)	Blood, saliva, feces, hair; interpret by matrix (acute or chronic)	Collection can induce stress; strong circadian variation; laboratory cost	(5, 93, 96)
IGF-1	Reduction of liver synthesis under prolonged stress; impacts growth and lactation	150–300 ng/ml	< 100 ng/ml (prolonged stress)	Blood sample, laboratory assay (ELISA)	Influenced by nutrition, age, lactational phase; cost and need for laboratory	(79, 94)
T3	Regulates basal metabolism; decreases endogenous heat production	0.5–2.0 ng/ml	< 0.5 ng/ml (chronic stress)	Blood samples, laboratory tests	Requires laboratory and variation by nutrition	(56, 75)
T4	Regulates metabolism and thermogenesis; decrease protect from heat	45–120 ng/ml	< 45 ng/ml (chronic stress)	Blood samples, laboratory tests	Requires laboratory and variation by nutrition	(56, 75)
Glucose	Energy mobilization via cortisol and catecholamines; reflecting energy balance	45–75 mg/dl	< 45 mg/dl or major fluctuations	Blood (dot)—glucometer; laboratory	Influenced by diet, fasting, sampling; rapid variations	(94, 95)
NEFA	Increase in compensatory lipolysis; indicator of prolonged metabolic stress	0.2–0.6 mmol/L	>0.6 mmol/L	Blood (clinical chemistry)	Affected by diet and postpartum period	(79, 94)

bpm, beats per minute; HPA, hypothalamic-pituitary-adrenal axis; ELISA, enzyme-linked immunosorbent assay; NEFA, non-esterified fatty acids; IGF-1, insulin-like growth factor type 1; T3, triiodothyronine; T4, thyroxine.

Recent research indicates that dairy cattle in hot climate regions have a significant increase in respiratory rate when exposed to direct solar radiation and high humidity (75, 76). This response is a thermoregulation mechanism, but when prolonged, it leads to greater energy expenditure and a drop in productive performance (77, 78). In tropical conditions, this indicator becomes even more relevant due to the intensity and duration of critical periods (79).

New technologies, such as rumen sensors and monitoring collars, have allowed the continuous recording of internal parameters, such as rumen and eyeball temperature (75, 76). These data offer greater accuracy in the early diagnosis of heat stress and enable rapid interventions (80). However, high costs and the need for connectivity limit the application in small farms, where direct observation of clinical signs, such as wheezing and excessive salivation are still prevalent (75, 81).

**Figure 3:**
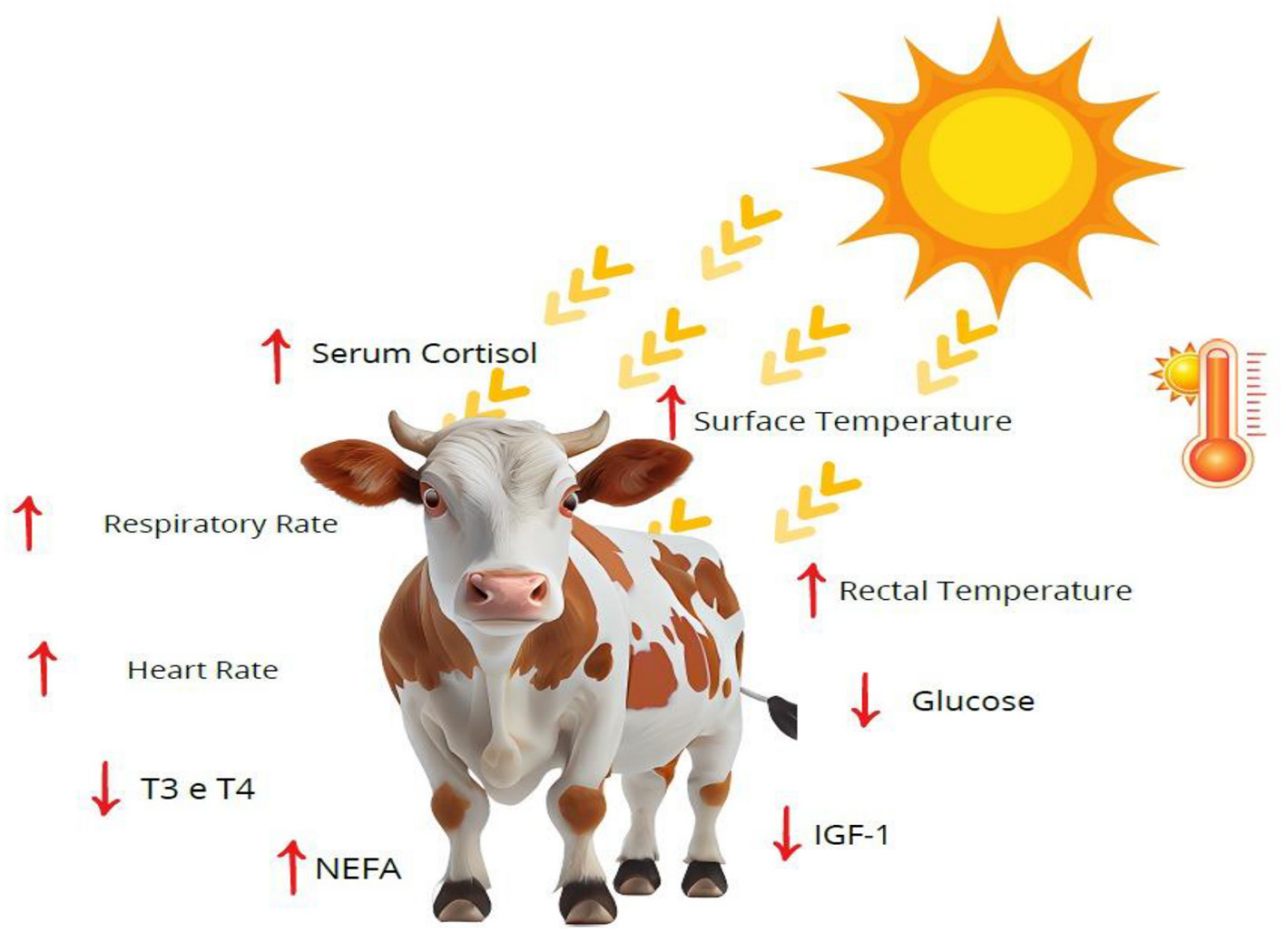
Impact of heat stress on the physiological and metabolic parameters of cattle. Authorship 2025. NEFA, non-esterified fatty acids; IGFT-1, insulin-like growth factor type 1; T3, triiodothyronine; T4, thyroxine.

Obtaining the results of these indicators is essential to understand the impact of heat stress on cattle (57). These parameters provide objective information about the body's adaptation to environmental conditions, and are crucial in tropical regions where temperature and humidity variations are intense (Figure 3) (15, 40).

Heart rate (HR) ranges between 60 and 80 bpm in adult cattle at rest, values above 90 bpm indicate heat stress, pain, or metabolic disorders (6, 12, 79). The increase in HR represents the intensification of peripheral circulation, aiming to increase heat dissipation (63, 74). Studies in grazing systems show that animals of lower social hierarchy tend to have higher HR in response to heat (82, 83).

HR reflects the activity of the autonomic nervous system and the balance between the sympathetic and parasympathetic branches (84). During heat stress situations, HR increases due to activation of the sympathetic system, which promotes peripheral vasodilation and increases cardiac output to dissipate heat (6, 7). Persistently high values indicate continuous effort by the body, which can lead to cardiovascular fatigue, reduced food intake, and a drop in productivity (79).

Respiratory rate (RR) is highly sensitive to thermal changes, cattle in comfort have 24–36 movements/min, while 60–80 movements/min indicate moderate discomfort and >100 movements/min, severe stress (14, 64, 82). The increase in RR results from the need for greater gas exchange to dissipate evaporative heat in the upper airways (72).

The increase in RR is reflected in the direct response to the need for evaporative heat dissipation in the airways, which activates respiratory centers in the medulla oblongata and medulla, raising RR to increase gas exchange and maintain homeothermy (47, 63). Prolonged high frequencies can induce hyperventilation, dehydration, and reduced feed efficiency, negatively impacting reproductive and productive performance (75, 77).

Rectal temperature is the main indicator of core temperature (85). Normal values are between 38.0 °C and 39.3 °C; readings above 39.5 °C suggest hyperthermia, while persistent temperatures above 40 °C reflect intense stress and impaired milk production (58, 72, 75). Studies in the Western Amazon show that prolonged exposure to the sun significantly increases rectal temperature, affecting feed intake and productive efficiency (4, 56).

Being a direct indicator of internal heat load, rectal temperature, at the time when the animal is under heat stress, endogenous thermogenesis increases, while heat dissipation mechanisms (respiration, transpiration, conduction, and radiation) try to compensate (40, 58). Higher-than-normal temperatures indicate that heat exchange capacity is insufficient, affecting metabolism, feed intake, and lactation (14, 67).

Surface temperature can be obtained by infrared thermography or digital surface thermometers, both used to assess thermolysis and identify hot areas related to heat stress, such as the eye, muzzle, and udder. In cattle, normal values range from 34 °C to 36 °C, while temperatures above 38 °C indicate acute stress (47, 63, 86). Infrared thermography, already established as a non-invasive tool, allows mapping of body heat distribution and identification of areas of increased heat without restraining the animal (3, 86), although it is sensitive to environmental interference, such as solar radiation, wind, and humidity, requiring standardization of measurements (87, 88).

**Figure 4:**
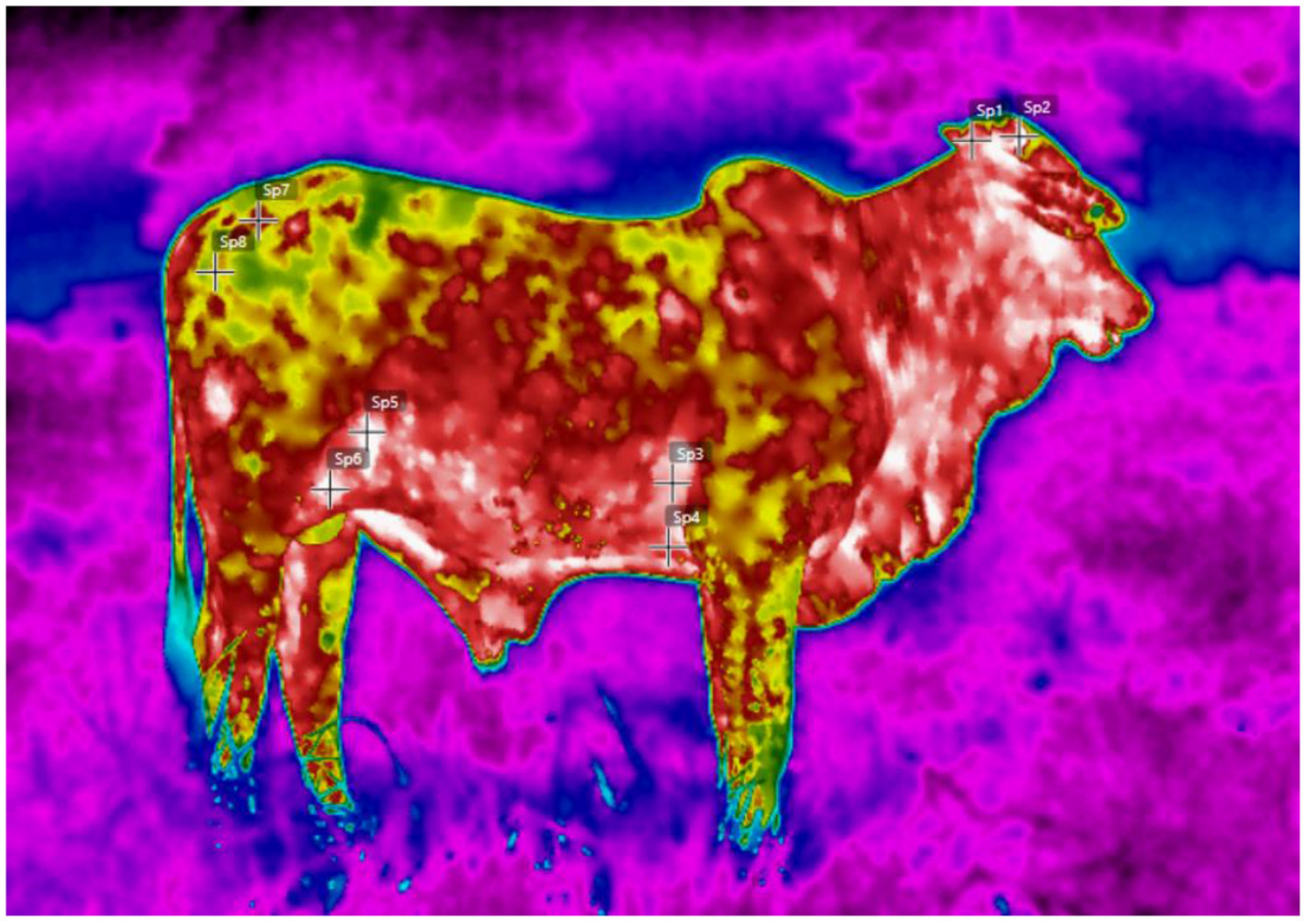
Applicability of infrared field thermography. Measurements of the anatomical regions: SP1 and SP2—head; SP3 and SP4—armpit; SP5 and SP6—flank; and SP7 and SP8—rump.

The digital surface thermometer, in turn, offers quick and simple measurements in the field and is less sensitive to environmental changes, but it only captures the temperature at the point of contact and can cause minor thermal changes or stress during handling (4, 81, 89). Thus, changes in surface temperature patterns—reflecting peripheral vasodilation during heat—can be detected early (86, 90), and the combination of thermography and surface thermometry provides a more robust assessment of the thermal status and comfort of animals under practical management conditions (91) (Figure 4).

Hormones, on the other hand, also provide important information about physiological status (92). Serum cortisol has reference values of 1–6 μg/dl, with levels >10 μg/dl are associated with acute stress (93, 94). IGF-1, being a marker of growth and reproduction, has normal levels of 150–300 ng/ml, reducing below 100 ng/ml in prolonged stress (95, 96). It is the thyroid hormones T3 (0.5–2.0 ng/ml) and T4 (45–120 ng/ml) that decrease in chronic stress, reflecting metabolic adaptation (22, 78).

Cortisol is the main glucocorticoid hormone released in response to stress, acting on energy metabolism, promoting gluconeogenesis and mobilization of fat and protein reserves (93, 94). Elevated cortisol levels reflect on the activation of the hypothalamic-pituitary-adrenal (HPA) axis and are associated with some behavioral changes, immunosuppression, and reduced production efficiency, constituting a reliable marker of acute and chronic stress (97).

Insulin-like growth factor 1 (IGF-1) is sensitive to nutritional status and stress load, where situations with high temperatures lead to prolonged stress, liver synthesis, and decreased IGF-1, affecting growth, reproductive efficiency, and milk production (95, 96). Its evaluation allows the identification of subclinical metabolic impacts, often not perceptible by behavioral indicators (98).

The thyroid hormones T3 and T4 regulate basal metabolism and thermogenesis, under heat stress, there is a reduction of these hormones to decrease energy metabolism and reduce endogenous heat production (22, 90). This metabolic adaptation helps with survival, but can compromise growth and milk production in prolonged situations (94).

There is also glucose, a relevant metabolic marker, which in adult cattle has normal values between 45 and 75 mg/dl (99). In conditions of intense excessive heat, levels can vary significantly, due to energy mobilization and hormonal changes, which can be associated with reduced food intake and reproductive changes (14, 70, 100). The combination of glucose with non-esterified fatty acids (NEFA) makes it possible to distinguish heat stress from concomitant pathological processes (101).

A metabolic marker sensitive to energy mobilization, glucose, during stress, there is an increase in cortisol and catecholamines, promoting gluconeogenesis and glycogenolysis, maintaining blood glucose levels (70, 100). Persistent fluctuations indicate energy imbalance and can interfere with milk production, reproduction, and immunity, reflecting directly on the animal (102). Non-esterified fatty acids (NEFA) increase in parallel with glucose, as a consequence of lipolysis to provide alternative energy, high values are indicative of prolonged stress and can predispose to metabolic disorders, such as ketosis, reflecting on health and productivity (103).

In summary, the integrated assessment provides a complete view of the state of the cattle, combining cardiovascular, respiratory, thermal, and hormonal parameters allows for a more accurate and grounded diagnosis to intervene in management, which favors and minimizes the effects of heat stress on health and productivity (37, 67, 77).

#### 3.3.2 Behavioral indicators

These are accessible and easy to interpret, offering a practical tool for producers, among the most observed indicators are the increase in standing time, the reduction of grazing, grouping, the search for shade and the proximity to drinking fountains (104). These responses represent natural heat dissipation strategies, but they reduce feed efficiency and compromise long-term productivity (7, 82).

Studies show that dairy cows exposed to moderate heat decrease forage intake time and increase permanence in shaded areas, when available (57, 78, 94). However, in tropical systems where native vegetation has been removed to form pastures, the lack of natural shade is detrimental, as it limits the main form of behavioral adaptation (22, 93).

**Figure 5:**
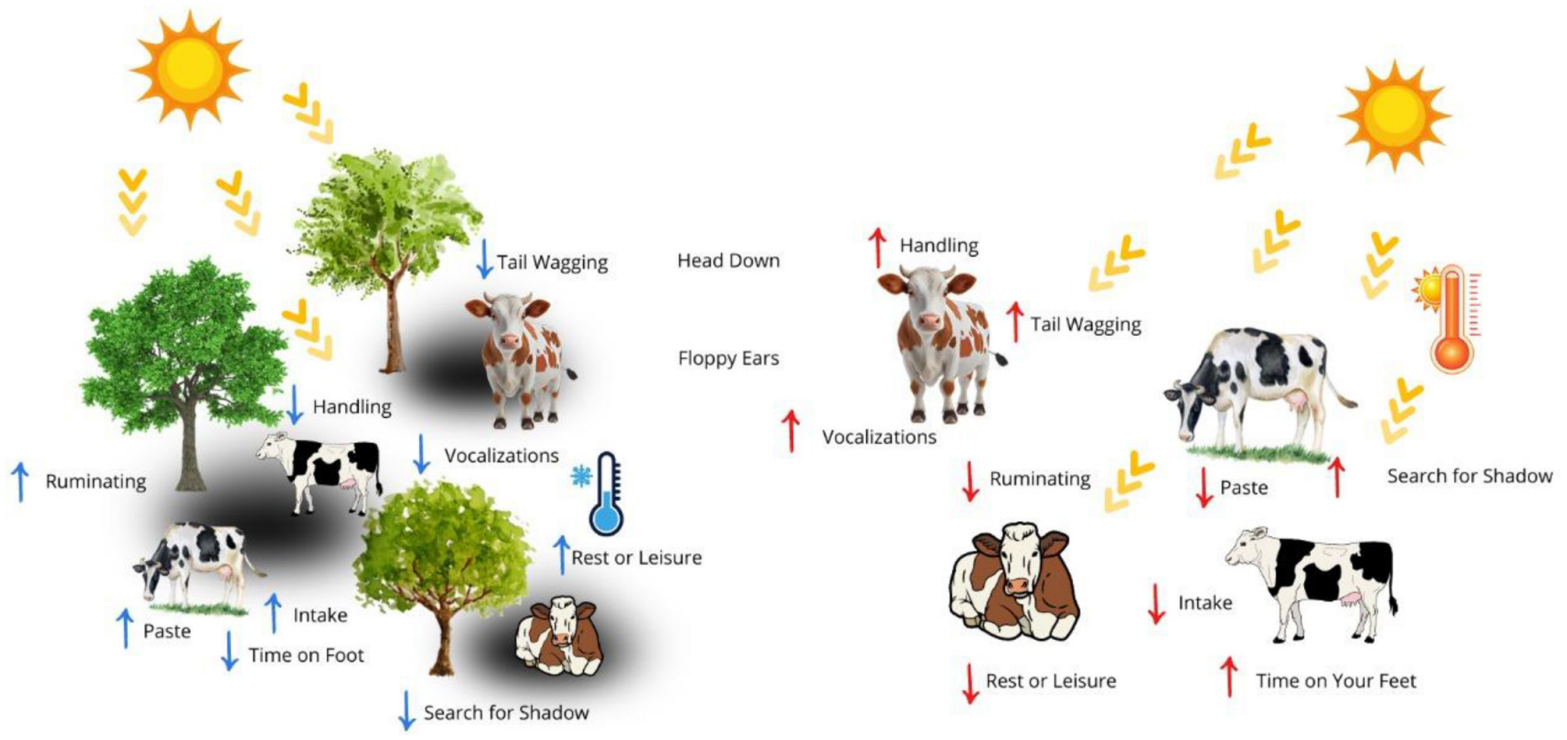
Behaviors expressed in relation to the environment. Authorship 2025.

Technological tools, such as collars with accelerometers and activity sensors, have been used to identify subtle changes in rumination and locomotion, allowing for early detection of animals that are more sensitive to heat (76, 105). Therefore, in smallholder systems, routine observation remains an effective strategy to identify signs of thermal discomfort (Figure 5) (6, 71).

Abnormal behavior of cattle is one of the first signs of changes in comfort and thermal balance, reflecting physiological adjustments and monitoring strategies (106). One of the most used parameters is the proportion of time standing and lying down, which in conditions of thermal comfort, remains about 60% of the day lying down and 40% standing (107). The increase in standing time, especially in the hottest hours, indicates discomfort and additional effort to dissipate heat, which can be obtained by direct observation, video cameras or position sensors (71, 82, 108).

The time of rest or idleness, usually 8–12 h/day in adult cattle, is essential for energy recovery and maintenance of rumination, another relevant indicator is the grouping and search for shade (109). Changes in these parameters reflect heat stress, pain, or metabolic discomfort, directly affecting digestive efficiency (6, 70). Rumination, which occurs between 7 and 10 h/day, is another important parameter; significant falls in this time or frequent interruptions indicate environmental or physiological malaise (37).

Changes in eating patterns are also clear indicators of discomfort, in comfort they dedicate 8–10 h/day to grazing, while intake drops significantly when the temperature exceeds the normal zone (64, 110). Reductions of more than 10% in habitual food consumption are characterized as a negative impact on digestion and energy balance (64, 79). In addition, the increased proximity to drinking fountains, usually visited 3–5 times/day, signals greater water demand and the search for body cooling (81, 94).

Other parameters, tail wagging, bowed head posture, floppy ears, excessive movement, and vocalizations showing a subtle increase in signs, may indicate irritation, thermal discomfort, or pain (111). Although these behaviors may seem like mild changes, they are considered early markers of stress (Table 2) (37, 93, 108, 112).

**Table 2:** Behavioral indicators.

**Indicator**	**Measurement method**	**Normal/standard values**	**Indicative of stress/discomfort**	**Limitations**	**Advantages**	**Citations in papers**
Time standing/lying down	Direct observation, cameras, position sensors	40% standing/60% lying down	Increased time on your feet, especially during hot hours	Influenced by herd management and routine	Easy monitoring; detects thermal discomfort	(3, 6, 82, 93)
Idleness/rest	Direct observation, activity sensors	8–12 h/day	Significant reduction in rest time	Can be confused with normal sleep	Early indicator of heat stress	(6, 82, 96)
Grazing/intake reduction	Observation, power and motion sensors	Low to moderate frequency	Reduced grazing time and intake	Influenced by nutrition and pasture availability	Reflects the impact of heat on productivity	(6, 45, 56, 82)
Tail swing	Direct observation	8–10 h/day	Increased frequency, especially with heat or irritation	Not very specific; requires interpretation	It is related to environmental stress and insects	(6, 7, 82)
Rumination	Observation, jaw sensors, devices	7–10 h/day	Reduction or frequent interruptions	Cost of sensors; complex interpretation	Reflects digestive wellbeing and thermal comfort	(3, 6, 71, 82)
Head posture, ears, and vocalizations	Direct observation, cameras, algorithms	Head and ears up, natural posture, occasional vocalizations	Head down, ears down, frequent vocalizations	Requires training and detailed analysis	Indicates pain, discomfort, social stress, allows continuous and remote monitoring	(6, 82, 94, 108)

In an integrated way, behavioral analysis provides valuable information about the response of animals to the environment (113, 114). Continuous monitoring, whether by direct observation or by sensor technologies, allows for early detection of uncomfortable situations, subsidizing interventions such as shading, ventilation, adjustment of animal density, and ensuring unrestricted access to water (77, 78, 96).

#### 3.3.3 Productive indicators

Productive indicators make it possible to quantify the economic impact of thermal discomfort, reflecting environmental conditions in results directly linked to production (47, 49, 63, 115). The reduction in dry matter intake is the main observation factor, reflecting in lower weight gain in beef cattle and in reduced milk production and quality in dairy cows (116, 117). These effects intensify in prolonged heat waves, which are common in tropical countries (Table 3) (51, 67).

**Table 3:** Productive indicators.

**Indicator**	**Evaluation method**	**Signs of normality**	**Changes indicative of stress**	**Recommended monitoring frequency**	**Citations in papers**
Average daily gain (ADG)	Periodic weighings, body score	Beef cattle: ADG ≥1.0 kg/day (depending on breed and diet)	Reduced weight gain or loss	Biweekly to monthly	(34, 47, 63, 66, 100)
Milk production	Daily records, tank or individual	Production according to genetic potential and diet	Decrease in daily volume and changes in total solids	Daily (ideal)/weekly (minimum)	(67, 75, 79, 96)
Milk quality	Laboratory fat, protein and SCC tests	Fat 3.5%−4.0%; protein 3.0%−3.5%; SCC < 200,000 cells/ml	Solids reduction; elevated SCC; risk of mastitis	Biweekly to monthly	(67, 75, 94, 96)
Heat expression	Visual observation, activity collars	Regular interval estrous cycles (18–24 days)	Reduction or absence of estrus; difficulty of detection	Monthly	(34, 79, 82, 95)
Conception rate	Reproductive records, gestational diagnosis	>40% in dairy cows; >60% beef cattle	Significant reduction in fertility; increase in services by design	By reproductive cycle; monthly analysis	(19, 34, 49, 95, 96)
Open days	Interval between delivery and new pregnancy	90–120 days in dairy systems	Delays of more than 150 days	Quarterly/semi-annual	(34, 93–96)

ADG, average daily gain; SCC, somatic cell count; cell, cells.

**Figure 6:**
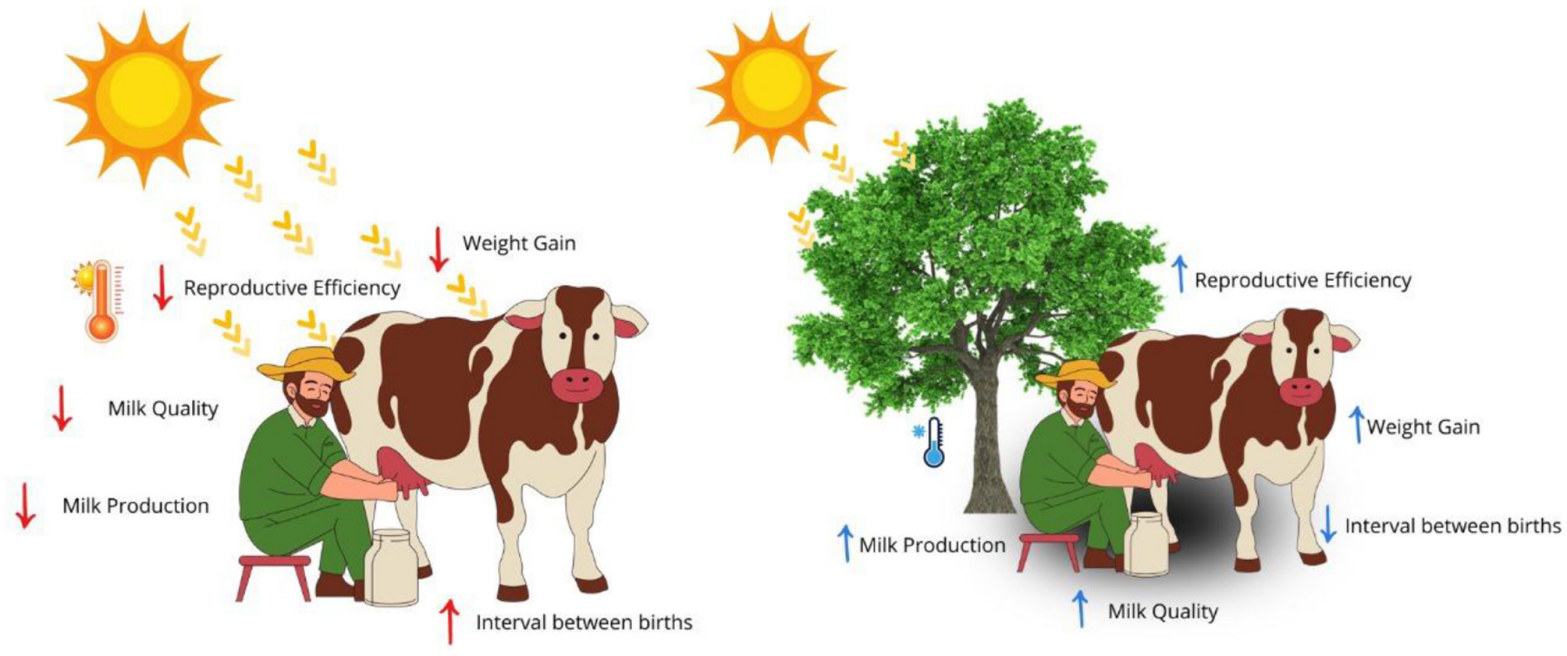
Impact of heat stress on the productive performance of dairy cows. Authorship 2025.

In addition to the drop in productivity, high temperature compromises reproductive efficiency, delaying the return to the estrous cycle and reducing conception rates, leading to a result of a longer calving interval and a decrease in the number of lactating cows, directly impacting the sustainability of the production systems (Figure 6) (28, 49). Extreme weather events, such as El Niño, have been associated with significant productivity losses, reinforcing the need for indicators for prevention (3, 4).

In practice, these indicators are valued by producers because they are directly linked to the profitability of the activity (118, 119). The combination of climate indices (such as THI), physiological parameters, and productive results allows for a more accurate assessment of risk and justifies interventions, from management adjustments to investments in cooling infrastructure (72, 74). This integration of measures is essential to strengthen the resilience of systems in the face of climate change (22, 77).

Environmental temperature directly compromises the productive and reproductive performance of cattle, diverting energy from growth and lactation to the maintenance of homeothermy (47, 63). In beef cattle, this is due to lower voluntary feed intake and a drop in conversion efficiency, reflected in a reduction in average daily gain (ADG) and lower muscle and adipose tissue deposition (34, 100). Monitoring should include periodic weighing, consumption records, and assessment of the body condition score, when persistent reductions in GDF and losses of body condition are indicative of warning (120, 121).

In dairy cattle farming, a drop in production is one of the most evident signs of heat stress, studies point to reductions in milk volume during periods of intense heat, especially in tropical systems, in addition to changes in total solids and fat/protein ratio (28, 67, 96). Monitoring should be daily, using individual or tank records, while periodic laboratory analyses evaluate composition and quality (49, 115).

Milk quality is also impacted, decreased fat and protein, and increased somatic cell count are associated with immunosuppression caused by heat, favoring greater susceptibility to mastitis (75, 94). Thus, the evaluation should include monitoring the composition of the milk and the health of the mammary gland, allowing for rapid preventive interventions (122, 123).

Heat stress delays the return to estrus and reduces fertility (124). The main effects are lower heat expression, increased open days, reduced conception rate, and greater number of services per conception (19, 95). Such changes result from hormonal failures, lower oocyte quality, and reduced embryonic viability (125).

Reproductive monitoring should include daily observation of estrus, insemination records, and pregnancy diagnoses (126). A drop in the conception rate by more than 10 percentage points or an increase in open days beyond the farm's goal should be considered warning signs (79, 94). These indicators provide essential information to identify the interference of the environment in the efficiency of the production systems.

Another relevant point is that the impacts of heat can have delayed effects, especially on reproduction. Changes in follicular and uterine circulation, even after the end of hot flashes, have repercussions on fertility for weeks (49, 93). This reinforces the importance of integrating productive and reproductive data into the climate context of each property (127).

Thus, it serves as central tools to assess animal welfare. Constant monitoring of milk production, composition, weight gain, intake, and reproductive parameters allows for early identification of risk situations and support management decisions (47, 63, 64, 110).

#### 3.3.4 Bioclimatic Indicators

Bioclimatic indicators are fundamental tools to analyze the relationship between the environment and the animal, making it possible to measure the degree of comfort or heat stress that cattle may be exposed to (12, 41, 128). These indices cover several climatic variables, such as air temperature, relative humidity, wind speed and solar radiation, as well as physiological variables, including rectal temperature, respiratory rate and black globe temperature, providing parameters that facilitate the detection of conditions potentially harmful to animal welfare (Supplementary Table S2).

### 3.4 Current technologies and their principles in management to optimize wellbeing

Cattle farming relies on the integration between monitoring technologies and appropriate management practices to assess welfare and heat stress (129). Heat stress, amplified by climate change, directly compromises the health, production, and reproduction of animals (130). Given this scenario, different electronic devices have been used to provide real-time data on the physiological and behavioral condition of cattle, helping producers to make decisions (56, 67).

One of the tools that has been gaining ground are rumen biocapsules, devices inserted in the rumen capable of continuously recording variables such as body temperature, pH, and rumen activity (131, 132). These sensors make it possible to identify metabolic and physiological changes resulting from heat stress at an early stage, correlating them with the temperature and humidity index (133). By sending information to software and applications, such technologies enable immediate adjustments in management (75).

Another widely used resource is smart collars used to assess rumination, frequency of food intake and swallowing time, locomotion activity, and time in season, helping to identify variations in behavior, such as increased idleness during heat waves or changes related to heat (6, 71).

Also, electronic devices integrated with cell phone applications and digital platforms allow the producer to monitor the data in real time (134, 135). This communication favors quick decisions, such as activating ventilation systems or transferring animals to shaded areas (112). In addition, artificial intelligence algorithms applied to these applications are able to predict risk scenarios, optimizing preventive management (108).

Infrared thermography, already consolidated in research, is also increasingly accessible in practice, and can be used in portable devices or attached to drones (136). Allowing to map surface body temperature and identify heat stress points in larger batches, reducing the need for individual containment (137). The associated use of thermal cameras and analysis software increases the accuracy in the characterization of the microclimate in facilities and pastures (4, 86).

These technological advances do not replace traditional management, but complement according to Bang et al. (67), Holinger et al. (82), Antanaitis et al. (6), and Nam et al. (75). Strategies such as natural and artificial shading, evaporative cooling, and silvopastoral systems remain key to reducing thermal load and providing greater comfort (3, 4). Innovation, in this sense, should be understood as a tool to support the sustainability of production, integrating science, wellbeing, and productivity (34, 79).

Finally, the role of bioclimatic zoning, associated with digital tools, as a medium and long-term planning strategy is highlighted (78). Climate modeling studies project risk scenarios for each region, allowing the adoption of adaptive practices before the effects of heat compromise the herd (49, 77, 78). Thus, the integration between biotechnologies, electronic devices, mobile applications, and appropriate management practices represents an irreversible trend for modern livestock, which seeks to reconcile animal comfort and production efficiency (81, 94).

### 3.5 Limitations and challenges

Despite the advances, the methodological difference in the diagnosis of heat stress remains high, from the selection of the index (THI, BGHI, IHT) to the cutoff points and outcomes (milk loss, physiological changes), which hinders and robust comparisons and synthesis in reviews (60, 67, 96). In tropical systems, this variability is critical due to the greater amplitude of humidity and radiation, little captured by models developed in temperate climates (22, 56).

Infrared thermography faces challenges of standardization (emissivity, distance, region of interest, time of day) and environmental interference (wind, dust, humidity), with an impact on repeatability between studies and farms (4, 86). In extreme events, such as El Niño 2023, transferring protocols between full sun and shaded systems requires local calibration to avoid false positives/negatives (3).

Behavioral sensors (rumination, locomotion, ingestion) show equipment and algorithm bias when applied to different and crossbred breeds, which are still underrepresented in validations, which limits accuracy for different biotypes and physiological stages (6, 37, 71). In grazing, noise due to heterogeneity of forage and topography reduces sensitivity to subtle changes in welfare (56, 82).

Rumen biocapsules expand internal monitoring, but the correlation between rumen temperature and external heat load varies with diet, passage rate and hydration; correction curves widely validated by climate/race are still lacking (75, 133). In addition, sensor drift and battery life require maintenance plans rarely described in field studies (6, 131).

AI/deep learning shows potential for prediction and screening (head/ear posture; imaging), but faces challenges of generalization across farms, cameras, and lighting conditions, as well as annotation bias and limited applicability to management decisions (138, 139). Performance reported in experimental data does not always translate into consistent gains in the commercial environment (108, 112).

There are social and hierarchical factors that can confound the effect of thermal interventions: social dominance alters access to shade, water, and trough, modulating physiological and behavioral responses and masking treatment effects (79). Bioclimatic zoning is advancing, but its spatial resolution and dependence on scarce weather stations generate uncertainty for property level decisions (64, 78). Climate projections bring divergent scenarios and require cross-validation with microclimate of silvopastoral facilities and landscapes (34, 77, 100).

In the link between wellbeing and productivity/reproduction, there is a lack of long time series that integrate sanitary and inflammatory events as modulators of heat responses (95). Immune challenged evidence indicates complex interactions between heat stress and inflammation that alter behavior and traditional wellbeing metrics, which are still poorly contemplated in reviews (94, 141–158). Regarding transferability between breeds and categories, *Bos indicus* and crossbred responses to stressors and mitigation differ from those of *Bos taurus*, requiring specific risk curves, especially for beef zebu and bulls (3, 93, 100). Calves continue to be studied in humid tropical climates, despite new non-invasive approaches with thermal signature (78, 140).

Adoption barriers persist at small and medium-sized properties: total cost of ownership, connectivity, interoperability between brands, and data governance (ownership, privacy, sharing) (63, 67). Even with access to pasture associated with behavioral benefits, the translation to simple operational metrics (e.g., management “triggers” via app) is still incipient (45, 81). Finally, there are measurement gaps: eye and body surface temperatures do not always reflect internal thermal load; multiple, standardized endpoints (physiological, productive, and behavioral) are required for integrated decisions (4, 81, 96). The combination of multimodal metrics and multicentric validation in real farm conditions is a differentiator for future syntheses.

Despite the advances discussed, this review presents some limitations and challenges that must be acknowledged. From a practical perspective, the applicability of welfare indicators and technological tools is constrained by factors such as high implementation costs, limited access to infrastructure, and the lack of shade and cooling strategies in extensive production systems, particularly in regions marked by socioeconomic inequalities among producers. These constraints may limit the adoption of advanced monitoring technologies, especially in small-scale or low-input systems, reinforcing the need for context-specific welfare assessment approaches.

From a methodological standpoint, this review is subject to limitations inherent to its design, including potential language bias, as only studies published in selected languages were considered, and database selection bias, which may have excluded relevant studies indexed elsewhere. In addition, differences in study designs, indicators assessed, and climatic conditions across regions may affect the comparability of results. These limitations highlight the need for future reviews to adopt broader search strategies and standardized assessment frameworks to strengthen the synthesis of evidence on cattle welfare under heat stress in climate change scenarios.

## 4 Conclusions

The present study demonstrates that the welfare of cattle is related to environmental conditions, being influenced by physiological, behavioral and productive indicators. The integration of these factors allows for a more accurate diagnosis of heat stress and supports management decisions that promote health, comfort, and productivity. Current technologies, such as sensors (rumen biocapsules, collars and ear tags) and thermography, complement direct observation, offering real-time data and enabling rapid interventions.

There are challenges such as costs, methodological heterogeneity and limitations of application in small systems that highlight the need for strategies adapted to the producer's reality. However, the combination of accessible practices and appropriate technologies represents a viable way to reconcile animal welfare, production efficiency and sustainability in modern cattle farming.

In addition, the evidence highlights the need to explicitly incorporate breed-related differences in thermal tolerance, particularly between European and Zebu cattle, into welfare assessment and management strategies aimed at improving resilience to heat stress. Future research should prioritize comparative and longitudinal studies that integrate genetic, environmental, and management factors, as well as the development of standardized protocols that enhance the applicability and reproducibility of welfare indicators across diverse production systems and climate change scenarios.

## Data Availability

The original contributions presented in the study are included in the article/Supplementary material, further inquiries can be directed to the corresponding author.
