# Supplementary material for: Welfare indicators in cattle farming in the face of heat stress: a review in climate change scenarios
Source: Front Vet Sci. 2026 Feb 11;12:1754412. doi: 10.3389/fvets.2025.1754412 (PMC12932167; doi:10.3389/fvets.2025.1754412)
Supplement: Supplementary file 1 [file Table_1.docx]

**Table S1** - Information on the articles used in the review.

| Cut Cattle Farming | | | | | | | |
| --- | --- | --- | --- | --- | --- | --- | --- |
| **Year** | **Authors** | **Title** | **Species** | **Breed** | **NA** | **well-being indicator** | **technology used** |
| [28] | Barreto  et al. | Thermal comfort and behavior of beef cattle in pasture systems monitored by visual observation and electronic device. | Cattle | Nelore | 64 | Two systems were used (Sol Pleno System and integrated Crop-Livestock-Forest system), evaluation of animal behavior, electronic monitoring, temperature indices of the black globe during two shifts of the day (morning and afternoon). | Weather station, accelerometer, acoustic sensor |
| [29] | Guimarães-Yamada et al. | Influence of Different Heat Stress Reduction Systems on Physiological and Behavioral Responses and Social Dominance of Holstein and Jersey Cows and Heifers on Pasture. | Cattle | Dutch / Jersey | 12 | The experiment used a 3×3 Latin square design, with a 3×2×2 factorial arrangement, evaluating three heat stress reduction systems (irrigation + shade, showers + shade and shade only), two breeds (Holstein and Jersey) and two physiological stages (lactating cows and pubertal heifers) | ¥¥ |
| [30] | Kumar  et al. | Study on the Adaptability of Livestock in the Central Plain Zone of Uttar Pradesh during the Spring and Summer Seasons | Cattle | Mestizo | 18 | The data were compared in two seasons of the year (spring and summer), collecting RT, RR, HR, THI. | Portable Weather Station |
| [4] | Silva  et al. | Characterization of Thermal Patterns Using Infrared Thermography and Thermolytic Responses of Cattle Raised in Three Different Systems during the Transition Period in Eastern Amazonia, Brazil | Cattle | Nelore | 30 | The following variables are measured: Meteorological, Physiological, RR, RT, Infrared Thermography. | Infrared Thermal Imaging (ITI) Camera** |
| [31] | Chapman  et al. | A deep learning model to predict cattle heat stress | Cattle | No Defined Breed | 200 | Behavioral data, duration of breathing, heat stress, meteorological evaluation, | HR-LDn Allflex eSense™, software Heatime® Pro +, SCR Engineers Ltd. |
| [32] | Romanello  et al. | Thermal comfort of Nellore (*Bos indicus*) and Canchim (*Bos taurus* x *Bos indicus*) bulls maintained in an integrated crop-livestock-forest system in tropical climate | Cattle | Nelore | 64 | The animals were separated into two systems (System without shading and integrated Crop-Livestock-Forest system), respiratory rate, blood samples (Cortisol and T3), thermographic image of the body surface. | Thermal imaging camera (Testo 890), automatic weather sensors (Apogee), automatic weather station (Campbell CR3000). |
| [33] | Romero et al. | Feasibility Study of Proposed Measures to Assess Animal Welfare on Zebu Cattle Farms within Pasture-Based Systems under Tropical Conditions | Cattle | Zebu Cattle | 788 | Management, health, and well-being were evaluated in each of the 24 properties, selected after completing a questionnaire | Observers |
| [15] | Arias and Mader | Evaluation of Four Thermal Comfort Indices and Their Relationship with Physiological Variables in Feedlot Cattle | Cattle | Angus  Hereford | 79 | Data on AT, RH, WS, Soil temperature, BST, TT, RR, THI were evaluated. | IButton loggers, weather station, anemometers. |
| [34] | Babola  et al. | The influence of the silvopastoral system on the physiological, behavioral and health responses of the Purunã breed of cattle | Cattle | Purunã | 30 | The following data were collected, division into two systems (Silvipastoral System and monoculture pasture system), collection of physiological data (Body temperature with infrared thermography), behavioral analysis, and parasitological examinations. | Infrared camera (FLIR**), portable weather station (THDLA-500**). |
| [22] | Ferreira  et al. | Impacts of climate change on livestock in Brazil | Cattle | ¥¥ | ¥¥ | Data were collected from multiple regions of the country, climate change in livestock (dairy cattle, beef cattle) and discuss possible response strategies, associated with animal comfort and welfare. | ¥¥ |
| [35] | Suhendro  et al. | Association of the heat shock protein 70.1 gene with the physiological and physical performance of Bali cattle | Cattle | Bali | 82 | Certain parameters, physiological characteristics (RR, HR, RT, were evaluated as indicators of the response to heat stress, WC, TC, , body condition score, and were also measured for physical performance. The measurements were carried out in a handling cage in the morning and afternoon. | PCR** |
| [3] | Silva  et al. | Thermographic Profiles in Livestock Systems under Full Sun and Shaded Pastures during an Extreme Climate Event in the Eastern Amazon, Brazil: *El Niño* of 2023 | Cattle | Nelore | 30 | Three traditional, silvopastoral and integrated livestock production systems were analyzed, with different types of shade and access to the bathing area, recording temperatures in different zones, such as areas with trees, pastures with forage and exposed pastures. | Infrared Thermal Imaging Camera (IRT**) |
| [7] | Idris  et al. | Behavioral responses of beef cattle to hot conditions | Cattle | Angus | 24 | Changes in the positioning of the main parts of the body (ear, tail and head), feeding behavior, body maintenance and respiratory dynamics were individually exposed to hot conditions and fed a cereal grain-based finishing diet. | Cameras (K-guard CW214H**; New Taipei City, Taiwan), Digital Video Recorder (LG**, XQ-L900H**; Yeouido-dong, Seoul, South Korea) |
| [36] | Moraes  et al. | Differences in the behavioral parameters of young zebu bulls and compounds maintained in non-forest systems or in integrated crop-livestock-forest systems | Cattle | Nelore / Canchim | 40 | Data were evaluated in two systems (pasture without shade and crop-livestock-forest system), behavioral evaluation (based on sensors), blood collection (cortisol evaluation), climatic variables. | Automatic weather station, electronic accelerometer sensors (C-Tech HealthyCow, CowMed Ltd.). |
| [14] | Silva  et al. | Thermal comfort of Nellore cattle (*Bos indicus*) managed in silvopastoral and traditional systems associated with rumination in a humid tropical environment in Eastern Amazonia, Brazil | Cattle | Nelore | 20 | Physiological, behavioral and environmental indicators were measured and analyzed. | Infrared Thermal Imaging Camera (IRT**) |
| [37] | Kim | Automated cattle head and ear pose estimation using deep learning for animal welfare research | Cattle | Japanese | 88 | The following data were measured based on anatomical characteristics: head and left ear. | WOULD |
| [38] | Monteiro  et al. | Enteric methane emissions and thermal comfort indices of Nellore steers in a livestock-forestry system in the Amazon biome | Cattle | Nelore | 24 | Data from different times of the year, meteorological evaluation, forage and methane emission of animals in the pasture are measured. | Automatic weather station, GreenFeed. |
| [39] | Singaravadivelan et al. | Evaluation of heat tolerance in Murrah buffaloes, crossbred Vechur cattle using the dairy search index | Cattle | Vechur | 12 | The following parameters were measured: RH, RR, HR, RT, twice a day. | heart rate monitor (PolarH10), |
| **Dairy Cattle Farming** | | | | | | | |
| **Year** | **Authors** | **Title** | **Species** | **Breed** | **NA** | **well-being indicator** | **technology used** |
| [40] | Yan  et al. | Evaluation of thermal indices as indicators of heat stress in dairy cows in temperate climate | Cattle | Jersey / Karan Fries | 24 | Physiological parameters (RT, RR, HR, BST, evaluation of climatic indices at different times of the year, infrared thermography) were measured. | Thermal Imaging Camera (FLIR** E60**), Portable Weather Station. |
| [41] | Mandal  et al. | Impact of environmental factors on physiological adaptability, thermal tolerance indices and yield in crossbred Jersey cows | Cattle | Jersey | 62 | The rearing environment (paddock, accommodation with concrete floor), AT, RH, THI, RT, RR, HR were evaluated. | ¥¥ |
| [42] | Soares et al. | Factors influencing reproductive performance in dairy cows | Cattle | ¥¥ | ¥¥ | The year of calving, the season of calving, stillbirth, retained placenta, the order of calving and the season of insemination, insemination season were analyzed. | ¥¥ |
| [43] | Bang  et al. | Application of infrared thermal technology to assess the level of heat stress and the reduction of milk production of cows in small tropical dairy farms. | Cattle | Holstein, Zebu, Jersey, Brown Swiss e Holândes | 344 | Direct indicators, such as age, lactation stage and body score, as well as indirect indicators, were evaluated through temperature measurements using infrared thermography | Thermal Imaging Camera |
| [44] | Oliveira et al. | Effects of the thermal environment on dairy cattle under grazing system in Western Amazonia, Brazil. | Cattle | Asian | 113 | DBT, RH, BGT, WS were measured to calculate THI, GTHI, RTL, RT, HR and RR. | ¥¥ |
| [45] | Vaidya  et al. | Comparative efficacy of three different heat tolerance indices for thermoadaptability during heat stress in cattle at the Livestock Farm Complex, Veterinary Extension Education. | Cattle | Deoni Cow | 24 | RT, RR, HR are monitored twice a day, and meteorological evaluation is evaluated. | Weather Station. |
| [46] | Bryant  et al. | Development of a thermal load index for dairy cattle in grazing | Cattle | Holstein  Friesian | 346 | Breeding systems, RR, climatic data (RH, THI, WS) are measured. | Automatic weather station |
| [47] | Ranzato et al. | Sensor-based behavior patterns can identify heat-sensitive lactating dairy cows. | Cattle | Holstein  Friesian | 369 | Climatic conditions, cow behavior, chewing behavior, activity time, head movement were monitored | Accelerometer, ear tag, weather station. |
| [6] | Antanaitis et al. | Evaluation of Rumination, Feeding and Locomotion Behavior During Heat Stress in Dairy Cattle Using Advanced Technological Monitoring | Cattle | ¥¥ | 9 | The experiment used the following parameters: Temperature and Humidity Index, Rumination, Feeding and Locomotor Activities. | RumiWatch, SmaXtec |
| [48] | Mincu  et al. | Infrared thermography as a non-invasive method for stress assessment in lactating dairy cows during isolation challenges | Cattle | Holstein Friesian | 20 | Evaluative data such as orbital and nasal temperatures, isolation challenge, suggesting approaches to assess social stress in cattle, stress in breeding ruminants, | Infrared Thermal Camera (FLIR**ONE** Pro LT**) |
| [49] | Andrade et al. | Zoning of the effects of heat stress on dairy cows in southeastern Brazil | Cattle | ¥¥ | ¥¥ | Meteorological data (AT and RH) were used, evaluation of economic losses from milk production | Automatic weather stations |
| [50] | Holinger et al. | Behavioral changes in moderate heat load in grazing dairy cows under farm conditions. | Cattle | ¥¥ | 57 | Climatic conditions (temperature and humidity), direct behavior of cows every 10 minutes, individual activity by accelerometers and interindividual distances via GPS were monitored during 30 days of study | GPS |
| [51] | Lovarelli et al. | Effect of prolonged heat stress in dairy cows on productive and behavioral traits | Cattle | Holstein Friesian | 1600 | Behavioral traits, daily milk production, average milking time, somatic cell count, fat percentage, protein percentage and lactose percentage were analyzed. Heat stress was analyzed considering the THI on average in 28 different time windows of continuous heat stress. | *NASA / POWER *** |
| [52] | Pichlbauer  et al. | Evaluation of different sensor systems to classify the behavior of dairy cows on pasture | Cattle | Simmental | 12 | The following data were evaluated: THI, milk production, feed intake, AT, dry matter intake, behavior. | Electronic activity sensors, robotic milking. |
| [53] | Crump et al. | Access to pasture and ocular temperature in dairy cows | Cattle | HolsteinFriesian | 32 | The parameters ocular temperature were evaluated, taking into analysis, the angulation, and distance. | FLIR** E8** Infrared Camera (FLIR** Systems, Kent**) |
| [54] | Nam et al. | Effect of Temperature-Humidity Index on Dairy Cow Productivity and the Correlation Between Temperature-Humidity Index and Rumen Temperature Using a Rumen Sensor | Cattle | Holstein Friesian | 125 | Data on temperature, humidity, milk yield, milk components, blood components and rumen sensor data were measured. | Rumen insert sensor (pH, Smaxtec plus cake) |
| [55] | Silva  et al. | Evaluation of physiological parameters of crossbred dairy cows maintained in Equatorial Amazonian climate in Western Amazonia | Cattle | ¥¥ | ¥¥ | AT, RH, Temperature and Relative Humidity, RT, HR, RR, BST and body surface temperature were measured. | ¥¥ |
| [56] | Liu  et al. | Monitoring, modelling and mitigating heat stress in dairy cattle building in Reading, UK: Impacts of current and projected heatwaves | Cattle | ¥¥ | ¥¥ | The effectiveness of building adaptations in specific spaces, such as cattle housing and milking parlors, particularly under extreme climatic conditions | DesignBuilder, EnergyPlus, exauthor |
| [57] | Sousa et al. | Bioclimatic zoning and impacts of climate change on dairy farming in Maranhão, Brazil | Cattle | ¥¥ | ¥¥ | The following meteorological parameters were evaluated: Minimum temperature, Maximum temperature, Wind speed. | TerraClimate |
| [58] | Marins et al. | Effects of heat stress reduction on behavioral response in lactating dairy cows before and after an intramammary lipopolysaccharide infusion | Cattle | Dutch | 30 | The following parameters were observed in this study: individual milk production, air temperature and relative humidity were monitored every 15 minutes, vaginal temperature, breast biopsy, rest time in a given season (winter and summer). | ¥¥ |
| [59] | Sousa et al. | Non-invasive evaluation of thermal comfort in dairy calves based on thermal signature | Cattle | Dutch | 10 | The following data were measured and the following data were measured: body surface temperature, combined with environmental data, and environmental data were collected five times a day (from 6 a.m. to 10 p.m., every four hours) | Thermal Camera |
| [60] | Tresoldi  et al. | A Comprehensive Study of Respiration Rates in Dairy Cattle in Mediterranean Climate | Cattle | Holstein  Jersey | 406 | The following parameters are evaluated: RR, behavior (lying down or standing), AT, RH, WS, GTHI, age, productive status, milk production | Portable Weather Station, Dairy Comp 305. |

^1^Note: NA = Number of Animals; ¥¥ = Information not provided in the literature; THI = Temperature-Humidity Index; ** = Brand or model; AI = Artificial intelligence; GPS = Global Positioning System; DBT = dry bulb temperature; RH = Relative humidity of the air; BGT = Black Globe Temperature; WS = Wind Speed; GTHI = Globe Temperature and Humidity Index; RTL = Radiant Thermal Load; RT = Rectal Temperature; HR = Heart Rate; RR = Respiratory Rate; AT = Air Temperature; BST = Body Surface Temperature; TT = Tympanic Temperature; WC= Body Weight and TC = Thoracic circumference; GPS = Global Positioning System.
