# Supplementary material for: Welfare indicators in cattle farming in the face of heat stress: a review in climate change scenarios
Source: Front Vet Sci. 2026 Feb 11;12:1754412. doi: 10.3389/fvets.2025.1754412 (PMC12932167; doi:10.3389/fvets.2025.1754412)
Supplement: Supplementary file 2 [file Table_2.docx]

**Table S2** - Bioclimatic Indicators.

| **Authors** | **Index** | **Formula** | **Value range / Interpretation (Cattle)** | **Use** | **Citations in papers** |  |
| --- | --- | --- | --- | --- | --- | --- |
| Thom [129] | Temperature and Humidity Index (THI) | THI = DBT + 0.36 × DPT + 41.5 | ≤ 72 indicates no stress; 72–78 indicates mild stress; 79–88 indicates moderate stress; and 89–98 indicates severe stress | Evaluate thermal discomfort considering Temperature and Humidity | [85,4] |  |
|  |  |  |  |  |  |  |
|  |  |  |  |  |  |  |
| Buffington et al. [130] | Black Globe Temperature and Humidity Index (BGHI) | BGHI = BGT + 0.36 (DPT) + 41.5 | ≤ 74 thermal comfort situations; 75–78 alerts; 79–84 hazards; and ≥ 85 emergencies | Evaluate heat exposure considering Solar Radiation and Humidity | [14,38] |  |
|  |  |  |  |  |  |  |
|  |  |  |  |  |  |  |
| Benezra [131] | Benezra Thermal Comfort Index (BTHI) | BTHI = RT / 38.33 + RR / 23 | ≥ 2.0 indicates difficulty in the degree of adaptation of animals to the environment; < 2.0 indicates greater ease of adaptation | Quantify the thermal equilibrium of the animals from the physiological parameters | [89,30] |  |
|  |  |  |  |  |  |  |
|  |  |  |  |  |  |  |
| Moran et al. [132] | Environmental Stress Index (ESI) | ESI = 0.63 x DBT − 0.03 x RH + 0.002 x SR + 0.0054 (DBT × RH) − 0.073 (0.1 × SR) −1 | < 25 low risk and ≤ 25 to ≤ 33 moderates to high risk | Evaluate the climatic conditions that can cause thermal discomfort and stress to the animals | [86,133] |  |
|  |  |  |  |  |  |  |
|  |  |  |  |  |  |  |
| Baêta et al. [134] | Equivalent Temperature Index (ETI) | ETI = 29.83628 − 0.11519 × AT + 0.00059 × RH − 0.30525 × WS | < 18 to 27 absences of problems; and 28 to ≥ 32 cautions | Evaluate the thermal comfort of the animals by combining the main environmental values that influence the thermal sensation. | [40,135] |  |
|  |  |  |  |  |  |  |
|  |  |  |  |  |  |  |
| Rhoad [136] | Iberian Heat Tolerance Index (HTI Iberian) | HTI Iberian = 100 – 18 (RT − 38.33) | ≥ 100 indicates greater animal comfort; < 100 indicates a decrease in heat tolerance capacity | Quantify the impact of environmental conditions on the thermal well-being of animals | [41,45] |  |
|  |  |  |  |  |  |  |
|  |  |  |  |  |  |  |
| Gaughan et al. [137] | Thermal Load Index (TLI) | TLI _(TG ≤ 25°C)_ = 8.62 + (0.38 x RH) + (1.55 x AT) – (0.5 x WS) or ICT _(TG > 25°C)_ = 8.62 + (0.38 x RH) + (1.55 x BGT) – (0.5 x WS) + e(2.4- WS) | ≤ 70 indicates thermal comfort; 71-77 alert; 78-86 moderate heat stress; and > 86 severe thermal stresses | Analyze and quantify the actual heat stress to which the animals are exposed, being the most complete and fully representing the reality of the production systems | [15,138] |  |
|  |  |  |  |  |  |  |
|  |  |  |  |  |  |  |
|  |  |  |  |  |  |  |
|  |  |  |  |  |  |  |
|  |  |  |  |  |  |  |
| Esmay [139] | Radiant Heat Charge (RHC) | RHC = σ (MRT)4 | < 400 indicate comfort; 400-600 mild stress; 600-700 moderate stress and > 700 severe stresses | Measure the impact of solar radiation on the thermal comfort of animals, being especially applicable on days of intense radiation | [36,46] |  |
|  |  |  |  |  |  |  |
|  |  |  |  |  |  |  |
|  |  |  |  |  |  |  |
|  |  |  |  |  |  |  |
|  |  |  |  |  |  |  |
| Berry et al. [140] | Decline in Milk Production (DMP) | DMP = -1.075 - 1.736 x NMP + 0.02474 x NMP x THI | Desirable persistence: drop ≤ 8% per month after peak lactation; Moderate drop: between 9% and 12% per month; e Sharp drop: > 12% per month (Low persistence) | Quantify the reduction in milk production throughout lactation | [49,115] |  |
|  |  |  |  |  |  |  |
|  |  |  |  |  |  |  |
|  |  |  |  |  |  |  |
| Hahn and Osburn [141] | Reduced Food Consumption (RFC) | RFC = -28.23 + 0.391 x L | Dairy cows: they can reduce dry matter intake by 10 to 30% in situations of moderate to severe heat; beef cattle in feedlot: reductions vary around 15 to 25% under intense heat; and Heifers and Calves: the drop is usually smaller, but still significant (5 to 15%) | Assess how much animals reduced food intake in situations of stress or environmental change | [49,111] |  |
|  |  |  |  |  |  |  |
|  |  |  |  |  |  |  |
|  |  |  |  |  |  |  |
|  |  |  |  |  |  |  |
|  |  |  |  |  |  |  |
| Berry et al. [140] | Estimated Respiratory Rate (ERR) | EER = (5.1 x AT) + (0.58 x RH) - (1.7 x WS) + (0.039 x SR) - 105.7 | ≤ 60 indicates thermal comfort; 60-80 mild heat stress; 80-120 moderate heat stress; and ≥ 120 severe heat stress | Assess thermal comfort and animal welfare without having to measure respiration directly | [15,60] |  |
|  |  |  |  |  |  |  |
|  |  |  |  |  |  |  |
| Gaalass [142] | Gaalass Heat Tolerance Test (Gaalass HTT) | Gaalass HTT = 100-14 (RT -101) | ≤ 100 highly heat-tolerant animals; 85-99 animal with good tolerance; 70-84 medium tolerance; and < 70 low tolerance | Evaluates the ability of cattle to adapt to heat stress | [30,143] |  |
|  |  |  |  |  |  |  |
|  |  |  |  |  |  |  |
| Benezra [131] | Benezra coefficient of adaptability (BAC) | BAC = RT + RR / 38.33 + 23 | ≤ 2.0 animals considered well adapted to heat; and < 2.0 animals considered less adapted and sensitive to heat | Evaluates the adaptability of cattle to heat stress in tropical and subtropical regions | [35,41] |  |
|  |  |  |  |  |  |  |
|  |  |  |  |  |  |  |
| Thomas et al. [144] | Dairy Search Index (DSI) | DSI = L / Lmax x RTref / RT x FRref / RR | = 1 or > 0.8 animals well adapted and maintain milk production; 0.6-0.8 animal with moderate tolerance and slight reduction in production; and < 0.6 heat-sensitive animals and significant drop in production | Assess heat adaptability | [30,39] |  |
|  |  |  |  |  |  |  |
|  |  |  |  |  |  |  |
|  |  |  |  |  |  |  |
|  |  |  |  |  |  |  |
|  |  |  |  |  |  |  |
|  |  |  |  |  |  |  |
|  |  |  |  |  |  |  |
| McGovern e Bruce [145] | Heat Storage Calculation (∆HS) | ∆ HS = (3600 × Harm × A) / (Mv × Hb) | ≤ 0.5 thermal comfort; 0.5-1.0 mild heat stress; 1.0-2.0 moderate stress; and > 2 severe stresses | Measure rectal temperature variation during or after exposure to heat stress | [4] |  |

^1^Note: THI = Temperature-Humidity Index; DBT = Dry Bulb Temperature; DPT = Dew Point Temperature; BGHI = Black Globe Temperature and Humidity Index; BGT = Black Globe Temperature; BTHI = Benezra Thermal Comfort Index; RT = Rectal Temperature; RR = Respiratory Rate; ESI = Environmental Stress Index; RH = Relative Humidity; SR = Solar Radiation; ETI = Equivalent Temperature Index; AT = Air Temperature; WS = Wind Speed; ITC Iberico = Iberian Heat Tolerance Index; TLI = Thermal Load Index; e = Natural Logarithm Base; RHC = Radiant Heat Load; σ = Stefan-Boltzmann constant; MRT = Mean Radiant Temperature; DMP = Decline in Milk Production; NMP = Normal Milk Production; RFC = Reduced Food Consumption; ERR = Estimated Respiratory Rate; Gaalass HTT = Gaalass Heat Tolerance Test; BCA = Benezra Adaptability Coefficient; DSI = Dairy Search Index; L = daily milk production per animal; Lmax = maximum production of the animal under comfortable conditions; RTref = Rectal Temperature considered normal; ∆TR = Heat storage calculation; Harm = stored heat; A = animal surface area; Mv = body mass. Hb = specific heat of the animal.
